# Expression of the Inhibitory Receptor TIGIT Is Up-Regulated Specifically on NK Cells With CD226 Activating Receptor From HIV-Infected Individuals

**DOI:** 10.3389/fimmu.2018.02341

**Published:** 2018-10-10

**Authors:** Xiaowan Yin, Tingting Liu, Zhuo Wang, Meichen Ma, Jie Lei, Zining Zhang, Shuai Fu, Yajing Fu, Qinghai Hu, Haibo Ding, Xiaoxu Han, Junjie Xu, Hong Shang, Yongjun Jiang

**Affiliations:** ^1^Key Laboratory of AIDS Immunology of National Health and Family Planning Commission, Department of Laboratory Medicine, The First Affiliated Hospital, China Medical University, Shenyang, China; ^2^Key Laboratory of AIDS Immunology of Liaoning Province, The First Affiliated Hospital of China Medical University, Shenyang, China; ^3^Key Laboratory of AIDS Immunology, Chinese Academy of Medical Sciences, Shenyang, China; ^4^Department of Laboratory Medicine, General Hospital of Shenyang Military Command, Shenyang, China; ^5^Department of Clinical Laboratory, The Second Affiliated Hospital of Soochow University, Suzhou, China; ^6^Collaborative Innovation Center for Diagnosis and Treatment of Infectious Diseases, Hangzhou, China

**Keywords:** TIGIT, CD226, CD155, NK cell, HIV

## Abstract

Natural killer (NK) cells are important for maintenance of innate immune system stability and serve as a first line of defense against tumors and virus infections; they can act either directly or indirectly and are regulated via co-operation between inhibitory and stimulatory surface receptors. The recently reported inhibitory receptor, TIGIT, can be expressed on the NK cell surface; however, the expression level and function of TIGIT on NK cells during HIV infection is unknown. In this study, for the first time, we investigated the expression and function of TIGIT in NK cells from HIV-infected individuals. Our data demonstrate that the level of TIGIT is higher on NK cells from patients infected with human immunodeficiency virus (HIV) compared with HIV-negative healthy controls. TIGIT expression is inversely correlated with CD4^+^ T cell counts and positively correlated with plasma viral loads. Additionally, levels of the TIGIT ligand, CD155, were higher on CD4^+^ T cells from HIV-infected individuals compared with those from healthy controls; however, there was no difference in the level of the activating receptor, CD226, which recognizes the same ligands as TIGIT. Furthermore, TIGIT was found to specifically up-regulated on CD226^+^ NK cells in HIV-infected individuals, and either rIL-10, or rIL-12 + rIL-15, could induce TIGIT expression on these cells. In addition, high TIGIT expression inhibited the production of interferon-gamma (IFN-γ) by NK cells, while TIGIT inhibition restored IFN-γ production. Overall, these results highlight the important role of TIGIT in NK cell function and suggest a potential new avenue for the development of therapeutic strategies toward a functional cure for HIV.

## Introduction

Natural killer (NK) cells are a vital component of the innate immune system and play a major role in responses against tumors and viral infections (1, 2). The functions of NK cells depend primarily on the integration of signals from activating and inhibitory cell surface receptors (3, 4). In particular, inhibitory receptors have been identified as vital regulatory molecules in the human immune system, and multiple inhibitory receptors modulate the immune responses of NK cells. These inhibitory receptors can be divided into two types, depending on the ligands they recognize (5–7). One is the classical type, which includes killer cell immunoglobulin-like receptors and leukocyte immunoglobulin-like receptors, which typically recognize class I major histocompatibility complex (MHC-I) molecules (8, 9). The other type is a group of inhibitory receptors, which bind nectin and nectin-like adhesion proteins, rather than recognizing MHC-I; this group includes TIGIT (T cell immunoglobulin and ITIM domain), CD226 (DNAM-1), and CD96 (TACTILE) (3, 10–12).

Among these inhibitory receptors, TIGIT is a newly emerging member of the immunoglobulin receptor superfamily, which has important immune regulatory functions (6, 13–15). TIGIT consists of an immunoglobulin variable region (IgV)-like domain, a type I transmembrane domain, and a cytoplasmic tail, which contains an immunoreceptor tyrosine-based inhibitory motif (ITIM) and an immunoglobulin tail tyrosine (ITT)-like motif (13, 16–19). TIGIT is expressed on immune cells, such as NK cells, and effector and memory T cells (20, 21). Ligands of TIGIT include poliovirus receptor (CD155, PVR) and nectin-2 (CD112, PVRL2), expressed on antigen presenting cells (APCs), T cells, and tumor cells (13, 18, 20, 22); TIGIT binds CD155 with higher affinity than CD112 (20). CD226 receptors compete with TIGIT for binding to the same ligands, and mediate positive stimulatory signaling (23).

The proportion of TIGIT on healthy human NK cells is higher than that on other lymphocytes, suggesting that TIGIT may be particularly important for NK cell function (24). The functions of TIGIT on NK cells have been studied in some aspects. For example, TIGIT has been identified as a protective factor that facilitates liver cell regeneration by interfering with the interaction between NK cells and hepatocytes in a mouse model (14); the expression of TIGIT on murine NK cells is up-regulated during the early stages of acute viral hepatitis and negatively regulates NK cell activation (25); and the over-expression of TIGIT inhibits NK-mediated killing of the NK cell lymphoblastic leukemia/lymphoma cell line, YTS (20). Recently, Tian's team demonstrated for the first time that only TIGIT is associated with NK cell exhaustion, but not the other checkpoint molecules, and that blocking TIGIT could enhance the anti-tumor responses of NK cells in colon cancer (21). These results suggest that TIGIT is crucial to the immunological function of NK cells.

Human immunodeficiency virus (HIV) infection causes dysfunction of the innate and adaptive immune systems, leading to opportunistic infections or tumors (26–28). HIV infection disturbs NK cell function and the surface expression levels of some receptors (4, 29); however, little is known about the expression of TIGIT on NK cells or whether TIGIT contributes to regulation of human NK cell function during HIV infection.

This study is the first to investigate TIGIT expression and function in NK cells from HIV-infected individuals. Levels of TIGIT were found to be higher on NK cells from patients with HIV compared with healthy controls. TIGIT expression was inversely correlated with CD4^+^ T cell counts and positively correlated with plasma viral loads. Furthermore, our data demonstrate that TIGIT is specifically elevated on CD226^+^ NK cells, and can suppress the function of NK cells.

## Materials and methods

### Study participants

This study included a total of 92 individuals, among which 44 were HIV-infected and had never been treated with antiretroviral therapy (HIV); 48 were HIV-negative healthy controls (HC) with normal routine blood examination results and no diseases of the immune system. Patients with HIV were recruited at the Red Ribbon Clinic in the First Affiliated Hospital of China Medical University. All were male, with a median age of 39 years (range, 17–64 years), and had no apparent active opportunistic infections. Among healthy controls, 91.7% were male and the median age was 41 years (range, 18–67 years) (Table 1). All individuals signed informed consent forms approved by the Research and Ethics Committee of The First Affiliated Hospital of China Medical University (Shenyang, China) and the study was conducted according to the principles of the Declaration of Helsinki.

**Table 1:** Characteristics of study participants.

	**HIV (*N* = 44)**	**HC (*N* = 48)**	***^#^P-value***
*Age (years)	39 (17–64)	41 (18–67)	>0.05
Sex, M:F	44:0	44:4	>0.05
**HIV subtype (*****n*****)**			
CRF01-AE	39	NA	–
CRF07-BC	1	NA	–
CRF67-01B	1	NA	–
B	1	NA	–
ND	2	NA	–
*CD4^+^ T cell counts (cells/mm^3^)	312 (7–739)	811 (419–1156)	<0.05
*Plasma level of HIV RNA (copies/mL)	78590 (2990–387000)	NA	–

All HIV subjects were antiretroviral therapy-naïve; *Data presented as median (range); ^#^For comparison between HIV and HC; NA, not applicable; ND, not determined; M, male; F, female.

### Detection of TIGIT, CD155, and CD226 expression

Cell phenotyping was detected by flow cytometry using fresh peripheral blood mononuclear cell (PBMC) samples, which were isolated by Hypaque-Ficoll (GE Healthcare, Uppsala, SE) density gradient centrifugation. The expression of TIGIT and CD226 receptors on live NK cells were detected in PBMC samples using the following immune fluorescent antibodies: anti-CD3-peridinin chlorophyll protein (PerCP, clone SK7; BD Biosciences, San Jose, CA, USA), anti-CD56-phycoerythrin (PE)-Cyanin7 (Cy7) (clone B159; BD Biosciences), anti-CD16-APC-Cy7 (clone 3G8, BD Biosciences), anti-TIGIT-allophycocyanin (APC) (clone A15153G; Biolegend, San Diego, CA, USA), APC mouse IgG1 κ isotype control (clone MOPC-21; Biolegend), anti-CD226-fluorescein isothiocyanate (FITC) (clone TX25; Biolegend), FITC mouse IgG1 κ isotype control (clone MOPC-21; Biolegend), and Fixable Viability Stain 510 (BD Horizon™). CD155 ligand expression was also determined on live CD4^+^ T cells in PBMC samples, using the following immune fluorescent antibodies: anti-CD3-PerCP, Anti-CD4-APC-Cy7 (clone RPA-T4; BD Biosciences), anti-CD155-BV421 (clone TX24; BD Biosciences), BV421 mouse IgG1, κ isotype control (clone X40; BD Biosciences), and Fixable Viability Stain 510. The expression of TIGIT was analyzed on NK cell subsets categorized based on expression CD16 and/or CD56; total NK cells included all four NK subsets. Samples were analyzed using an LSR II Fortessa cytometer (BD Biosciences), which was adjusted with 10-peak color rainbow beads (Spherotech, Lake Forest, IL, USA). Between 300,000 and 500,000 lymphocyte events were collected for each sample. Gates were defined using corresponding isotype controls. The expression of each sample was analyzed by FlowJo 7.6 software (Ashland, OR, USA).

### Detection of interferon-gamma (IFN-γ) production and degranulation of NK cells (CD107a)

IFN-γ release and cytotoxic molecule degranulation (CD107a) levels were assessed by flow cytometry. For staining, PBMCs were seeded in 96-well round-bottom plates (50,000–60,000 per well) and stimulated with a cocktail of cytokines (IL-12, IL-15, and IL-18 at 10, 20, and 100 ng/ml, respectively; R&D Systems, Minneapolis, MN, USA). Unstimulated cells were used as negative controls. Cells were incubated in RPMI media containing 10% fetal bovine serum (FBS; Thermo Fisher Scientific, Waltham, MA, USA) and 1% penicillin/streptomycin (PS) for 24 h at 37°C with 5% CO_2_ in the presence of anti-CD107a-PE antibody (clone H4A3; BD Biosciences), in a total volume of 200 μL. GolgiStop (1 μL) (BD Biosciences) was added during the final 4 h of culture. Cells were harvested, washed, and stained with anti-CD3-FITC (clone SK7; BD Biosciences), anti-CD56-PE-Cy7 (clone B159; BD Biosciences), anti-CD16-APC-Cy7 (clone 3G8; BD Biosciences), and anti-TIGIT-PerCP/Cy5.5 (clone A15153G; Biolegend) in staining buffer on ice in the dark for 20 min. APC mouse IgG1, κ isotype control (clone MOPC-21; Biolegend), PE mouse IgG1, κ isotype control (clone MOPC-21; Biolegend), and PerCP/Cy5.5 Mouse IgG1, κ Isotype control (clone MOPC-21; Biolegend) were used to define gates. Cells were then fixed with Fixation/Permeabilization solution (BD Biosciences) at room temperature in the dark for 20 min, washed twice with Perm/Wash buffer (BD Biosciences), and intracellularly stained with anti-IFN-γ-APC (clone B27, BD Biosciences) for 20 min. Then cells were washed and re-suspended in staining buffer and analyzed in the BD FACS Canto II cytometer (BD Biosciences).

### Detection of changes in TIGIT expression levels on CD226^+^ NK cells treated with different stimuli

Changes in TIGIT expression levels on CD226^+^ NK cells were detected by flow cytometry. For staining, PBMCs were seeded in 96-well round-bottom plates (50,000–60,000 per well) and stimulated with IL-10 (10 ng/mL; R&D Systems), IL-12 + IL-15 (10 ng/mL and 50 ng/mL, respectively), or transforming growth factor-beta (TGF-β) (50 ng/mL; R&D Systems) respectively. Unstimulated cells were used as negative controls. Cells were incubated in RPMI media containing 10% fetal bovine serum (FBS; Thermo Fisher Scientific) and 1% penicillin/streptomycin (PS) for 24 h at 37°C with 5% CO_2_ in a total volume of 200 μL, then harvested, washed, and stained with anti-CD3-PerCP (clone SK7; BD Biosciences), anti-CD56-PE-Cy7 (clone B159; BD Biosciences), anti-CD16-APC-Cy7 (clone 3G8; BD Biosciences), and anti-TIGIT-APC (clone A15153G; Biolegend) in staining buffer on ice in the dark for 20 min. APC mouse IgG1, κ isotype control (clone MOPC-21, Biolegend) was used to define gates. Cells were then washed twice with PBS containing 2% FBS, re-suspended in staining buffer, and analyzed using a BD FACS Canto II cytometer (BD Biosciences).

### TIGIT, CD226, and CD155 blockade assays

The effects of blocking TIGIT, CD226, or CD155 on NK cell functions were assessed by preincubating PBMCs in the presence of functional grade purified anti-human TIGIT antibody (5 μg/mL; eBioscience, San Diego, CA, USA), purified anti-human CD226 antibody (20 μg/mL; Biolegend), purified anti-human CD155 antibody (20 μg/mL; Biolegend), or purified mouse IgG1, κ isotype control (5 μg/mL or 20 μg/mL; Biolegend) for 1 h before stimulation with IL-12, IL-15, and IL-18 (10, 20, and 100 ng/ml, respectively) for 24 h at 37°C and 5% CO_2_. After an incubation, IFN-γ release and cytotoxic molecule degranulation (CD107a) were evaluated as described above.

Recombinant human TIGIT Fc Chimera Protein (rTIGIT; 25, 50, and 100 ng/mL; R&D Systems) and LEAF™ Purified Mouse IgG1, κ Isotype control (25, 50, and 100 ng/mL; Biolegend) were added to pre-treated PBMCs for 1 h before stimulation with IL-12, IL-15, and IL-18 (10, 20, and 100 ng/ml, respectively) for 24 h at 5% CO_2_ and 37°C. IFN-γ release and cytotoxic molecule degranulation (CD107a) were evaluated as described above.

### Detection of absolute CD4^+^ T cell counts

Anticoagulant-treated whole blood samples (50 μL) and 20 μL TriTEST anti-CD4-FITC/CD8-PE/CD3-PerCP (BD Biosciences) reagents were added into Trucount tubes (Becton Dickinson). A single-platform lyse-no-wash procedure was performed and cells detected using a BD FACS Calibur flow cytometer. Data were analyzed using MultiSET software.

### Measurement of plasma HIV RNA levels

Reverse transcription polymerase chain reaction (RT-PCR) was used to determine plasma HIV RNA levels. The process was performed using the COBAS® AmpliPrep®/COBAS Taqman system (Roche Diagnostic Systems, Indianapolis, IN, USA); the detection range of this assay is 20–10,000,000 copies/mL. The manufacturer's reference standards were used to calculate HIV RNA copy number.

### Statistical analysis

All data were found to have nonparametric distributions on testing for normal distribution. The nonparametric Mann-Whitney U test was used for evaluation of differences in quantitative data between two groups, and the Kruskal-Wallis test employed for comparisons of multiple groups. The Spearman's rank test was used for analysis of correlation between two groups. A Wilcoxon matched-pairs test was used for paired group comparisons. *p*-values < 0.05 were considered statistically significant. All data analysis was performed using GraphPad Prism Version 6.0 software (GraphPad Software, La Jolla, CA, USA).

## Results

### The expression of TIGIT on NK cells is higher in individuals infected with HIV and correlates with HIV disease progression

**Figure 1:**
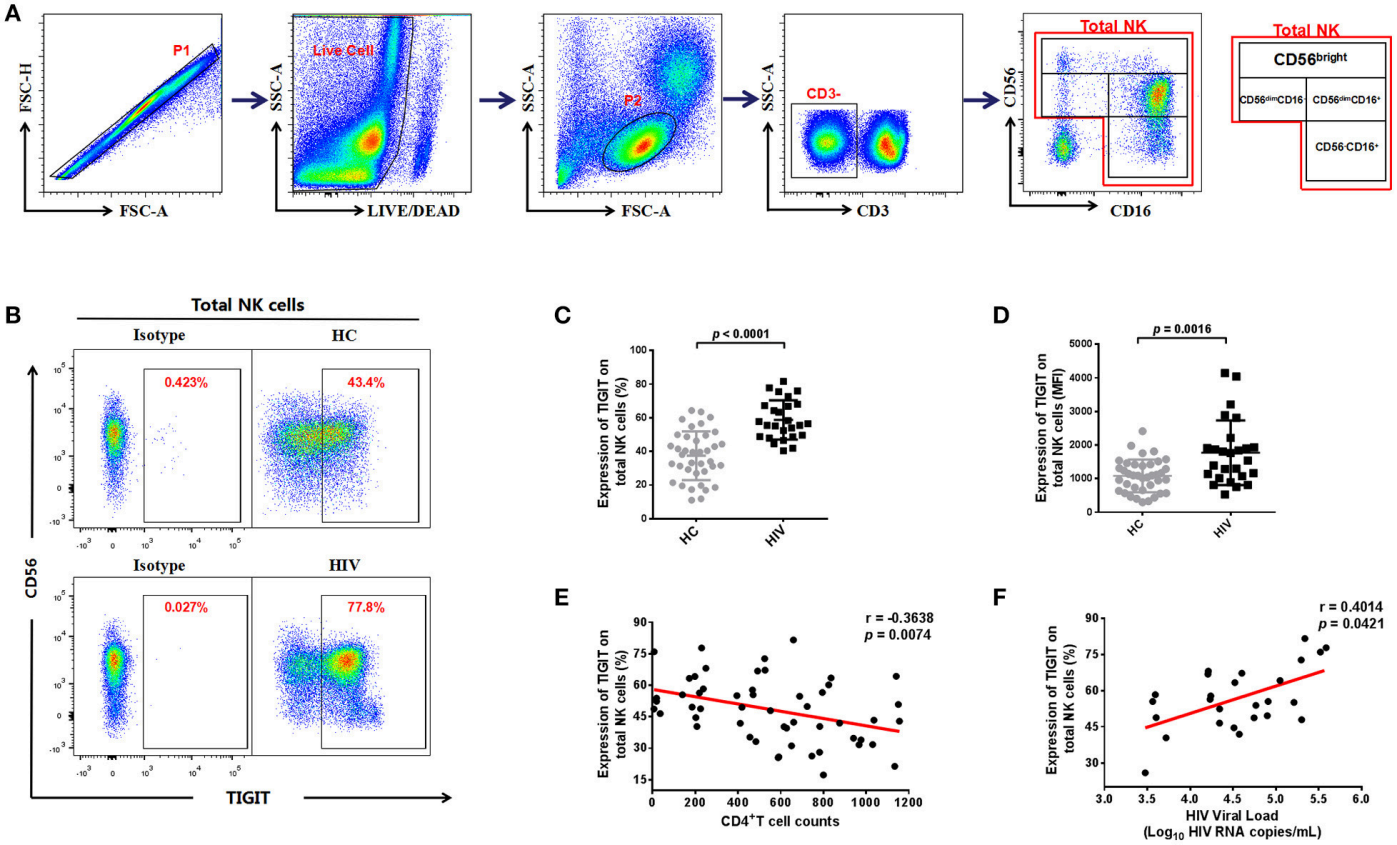
The expression of TIGIT on NK cells is higher in HIV-infected individuals and correlated with HIV disease progression. **(A)** Gating strategy used to identify total natural killer (NK) cells and NK cell subsets. Single cells were gated using the forward scatter area (FSA) and forward scatter height (FSH), then live cells were gated by Live/Dead (BV510) staining. Lymphocytes were gated according to forward scatter/side scatter properties (FSC/SSC). Total NK cells were identified from CD3-negative lymphocytes by their expression of CD16 and/or CD56. The four NK cell subsets identified were CD3^−^CD56^bright^CD16^−/+^, CD3^−^CD56^dim^CD16^+^, CD3^−^CD56^dim^CD16^−^, and CD3^−^CD56^−^CD16^+^. All the plots were based on an HIV+ individual. **(B)** A representative flow cytometry plot showing the different percentages of TIGIT on NK cells in the HC and HIV groups. The expression of TIGIT was gated according to an isotype control. **(C)** Comparison of the percentages of TIGIT on NK cells from the HIV (*n* = 26) and HC (*n* = 38) groups. **(D)** Comparison of the MFI of TIGIT on NK cells from the HIV (*n* = 26) and HC (*n* = 38) groups. **(E)** Analysis of the correlation between TIGIT expression on NK cells and absolute CD4^+^ T cell counts (cells/mm^3^) at the same sampling time (*n* = 53). **(F)** Analysis of the correlation between the proportion of TIGIT on NK cells and plasma levels of HIV RNA (Log–_10_ HIV RNA copies/mL) at the same sampling time (*n* = 26). A Mann-Whitney *U* test was used for comparisons between two groups. Error bars indicate the median and interquartile range. Spearman's rank analysis was employed for evaluation of correlation. *p* < 0.05 was considered significant.

We assessed the expression of TIGIT on NK cells from the HIV and HC groups by flow cytometry (representative plots are presented in Figures 1A,B and Figure S1). We found that TIGIT expression levels were significantly higher on total NK cells in the HIV-infected group than the HC group (*p* < 0.0001; Figure 1C), and the TIGIT mean fluorescence intensity (MFI) was also significantly higher in HIV-infected group compared to HC group (*p* = 0.0016; Figure 1D). NK cells can be divided into four distinct subgroups based on their surface expression of CD56 and CD16 (30), and the proportions of four NK cell subsets in our study participants were shown in Figure S2. We found the proportions of CD56^−^CD16^+^ NK subset was higher in HIV group (*p* = 0.0002); whereas, the proportions of CD56^dim^CD16^−^ NK subset was lower in HIV group (*p* < 0.0001). Next, we profiled TIGIT expression patterns in NK cell subsets from the HC and HIV groups. Representative flow cytometry plots are presented in Figure S3A with statistical analysis in Figures S3B,C. We found TIGIT levels were elevated in three NK subsets (CD56^−^CD16^+^, CD56^dim^CD16^−^, and CD56^dim^CD16^+^) in the HIV group (*p* < 0.0001, *p* = 0.0274, and *p* = 0.0800, respectively; Figure S3D). Conversely, TIGIT expression was relatively higher on the CD56^bright^CD16^−/+^ NK cell population in the HC group compared with the HIV group (*p* < 0.0001; Figure S3D).

Next, we analyzed the association of TIGIT expression levels on NK cells with CD4^+^ T cell counts and plasma viral loads. We found that the frequencies of TIGIT on total NK cells and four NK subsets were negatively correlated with CD4^+^ T cell counts (Total NK cells: *p* = 0.0074, *r* = −0.3638; Figure 1E; CD56^−^CD16^+^ NK: *r* = −0.5496, *p* < 0.0001; CD56^bright^CD16^−/+^ NK: *r* = −0.4079, *p* = 0.0024; CD56^dim^CD16^+^ NK: *r* = −0.2917, *p* = 0.0341; CD56^dim^CD16^−^ NK: *r* = −0.3205, *p* = 0.0193; Figures S3E–H); meanwhile, it was positively correlated with plasma viral loads (*p* = 0.0421, *r* = 0.4014; Figure 1F). These results indicate that the proportion of TIGIT on NK cells in HIV-infected individuals may be associated with disease progression.

### The expression of TIGIT limits the production of IFN-γ in NK cells

**Figure 2:**
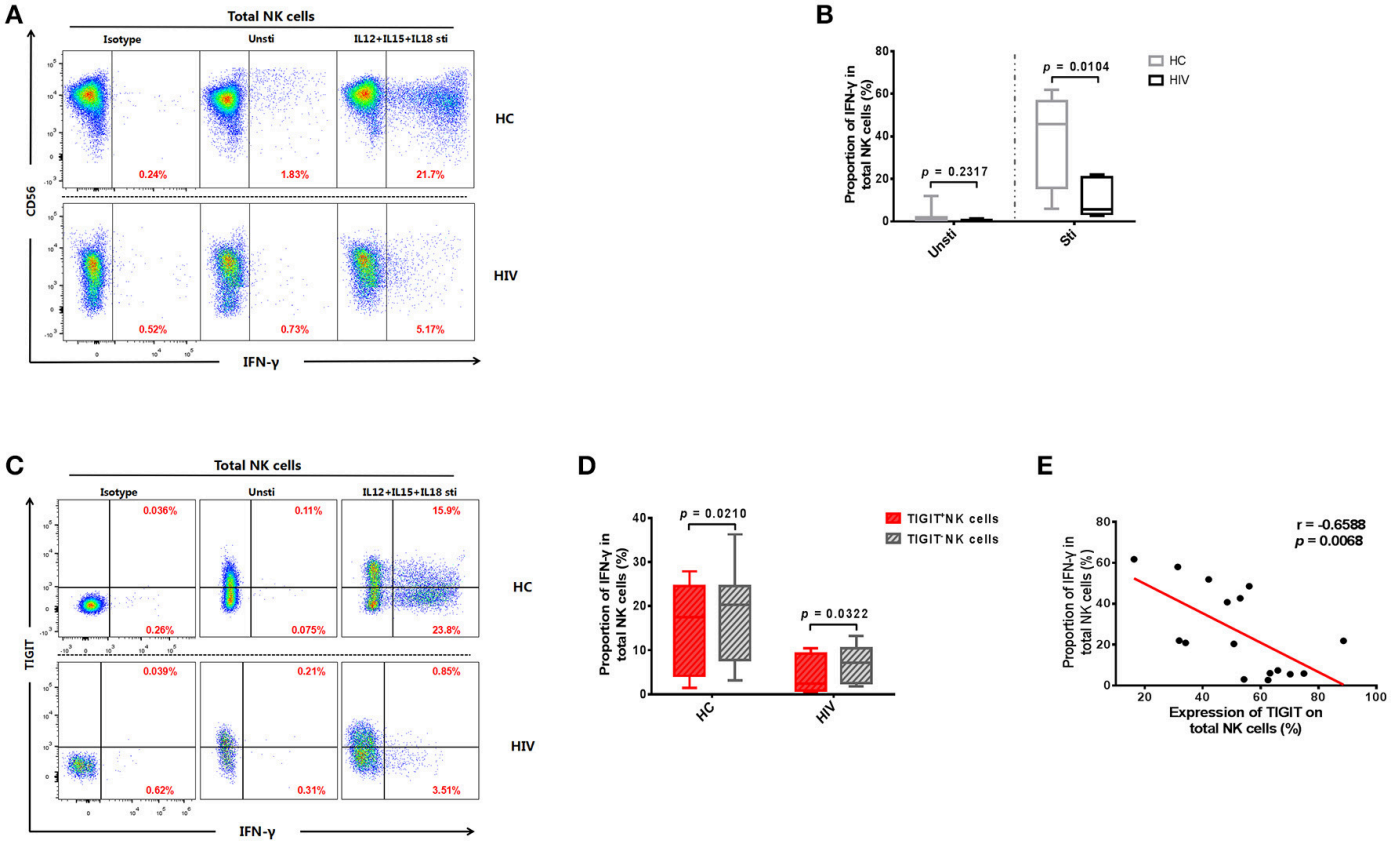
Expression of TIGIT limits the production of IFN-γ by NK cells. **(A)** A representative flow cytometry plot showing the different percentages of IFN-γ producing NK cells after stimulation with rIL-12 + rIL-15 + rIL-18 for 24 h in HC and HIV-infected individuals. The IFN-γ expression was gated according to an isotype control. **(B)** Comparison of the function of NK cells from HIV-infected (*n* = 8) and HC (*n* = 8) groups, based on their IFN-γ production on stimulation with rIL-12 + rIL-15 + rIL-18. **(C)** A representative flow cytometry plot showing the different percentages of IFN-γ producing TIGIT^+^ and TIGIT^−^ NK cells from HIV-infected individuals after stimulation with rIL-12 + rIL-15 + rIL-18 for 24 h. **(D)** Paired comparisons of IFN-γ producing TIGIT^+^ and TIGIT^−^ NK cells from HIV-infected individuals after stimulation with rIL-12 + rIL-15 + rIL-18 for 24 h (HIV: *n* = 11; HC: *n* = 12). **(E)** Analysis of the correlation between the percentage of TIGIT^+^ NK cells and the percentages of IFN-γ producing NK cells from HIV-infected individuals (*n* = 16). Mann-Whitney *U* tests were used for comparisons between two groups. The Wilcoxon matched-pairs signed-rank test was used for paired-group comparisons. The Spearman's rank test was employed for correlation analyses. *p* < 0.05 was considered significant.

HIV disease progression may be related to impaired NK cell function (31, 32). Therefore, we tested the levels of IFN-γ produced by NK cells from the HIV and HC groups. The results demonstrated that NK cells from HIV-infected individuals secreted less IFN-γ than those from the HC group (*p* = 0.0148; Figures 2A,B). Next, we explored whether NK cell IFN-γ production levels were affected by TIGIT expression. We observed a higher level of IFN-γ production in TIGIT^−^ compared with TIGIT^+^ NK cells in both the HIV and HC groups (*p* = 0.0322 and *p* = 0.0210, respectively; Figures 2C,D). Furthermore, there was a negative correlation between the percentage of TIGIT^+^ NK cells and IFN-γ^+^ NK cells (*p* = 0.0068, *r* = −0.6588; Figure 2E). This finding suggests that the presence of dysfunctional NK cells in HIV-infected individuals may be due (at least in part) to higher expression of the inhibitory receptor, TIGIT.

### Expression of the TIGIT ligand, CD155, is increased on CD4^+^ T cells from HIV-infected individuals

**Figure 3:**
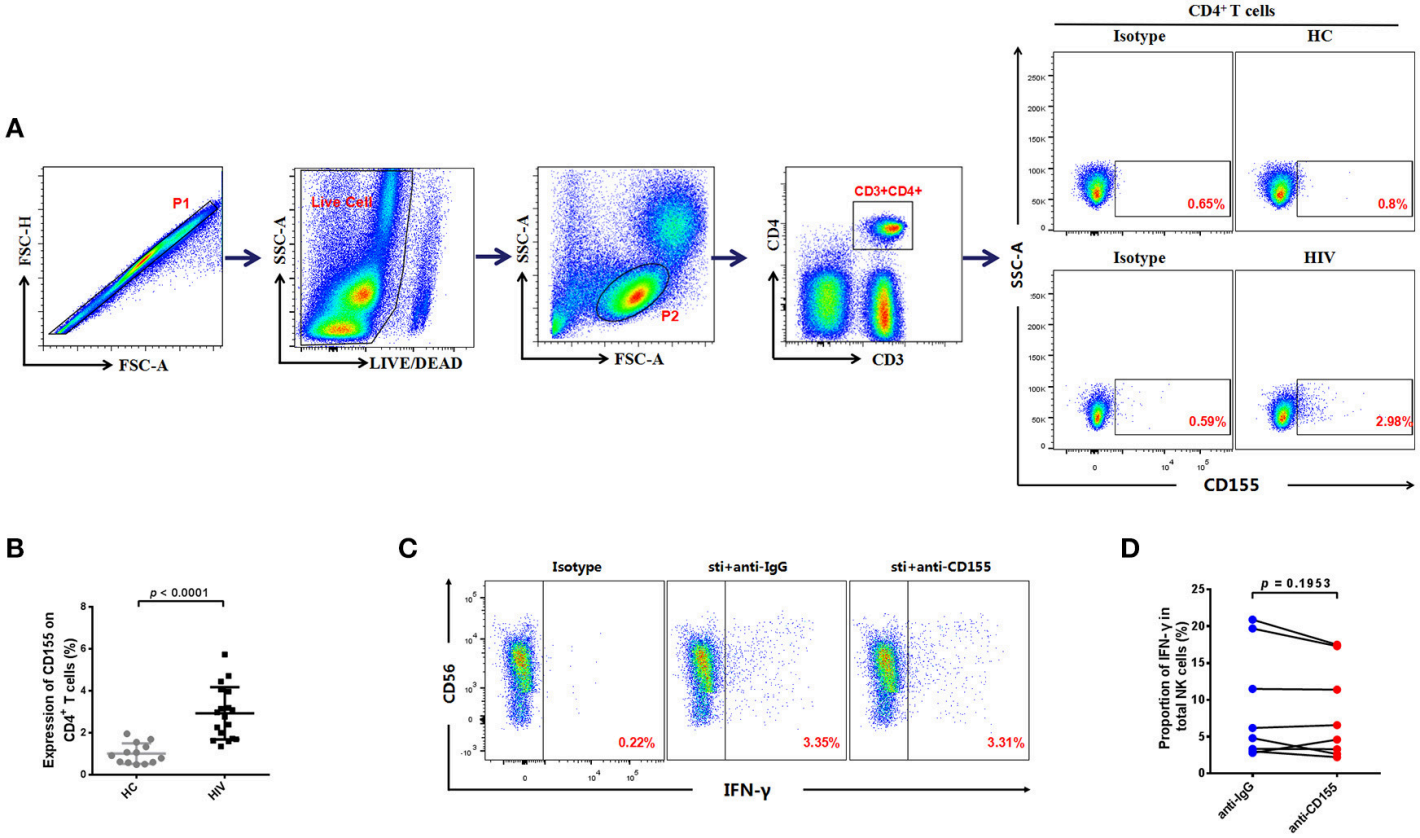
Association between the expression of CD155 on CD4^+^ T cells and IFN-γ production by NK cells from HIV-infected individuals. **(A)** Gating strategy used to identify the CD4^+^ T cells. Single cells were gated using the forward scatter area (FSA) and forward scatter height (FSH) (P1), then live cells were gated by Live/Dead (BV510). Lymphocytes were gated according to forward scatter/side scatter properties (FSC/SSC) (P2). CD4^+^ T cells were identified from the CD3^−^CD4^+^ gate. A representative flow cytometry plot showing the expression of CD155 on CD4^+^ T cells in HIV-infected and HC individuals. **(B)** Comparison of the percentages of CD155 expressing CD4^+^ T cells between HIV-infected (*n* = 18) and HC (*n* = 13) groups. **(C)** A representative flow cytometry plot showing production of IFN-γ in NK cells after treatment with 20 μg/mL anti-human CD155 antibody or purified mouse IgG (as a negative control) from HIV-infected individuals; the production of IFN-γ was gated according to an isotype control. **(D)** Paired comparison of the production of IFN-γ by NK cells after treatment with 20 μg/mL anti-human CD155 antibodies or purified mouse IgG (as a negative control) from HIV-infected individuals (*n* = 8). The Mann-Whitney *U* test was used for comparisons between two groups. The Wilcoxon matched-pairs signed-rank test was used for paired-group comparisons. *p* < 0.05 was considered significant.

TIGIT can exert its inhibitory function through binding with its ligand, CD155, which can be expressed on T cells (18); CD4^+^ T cells are the main target cells for HIV (33). Therefore, we evaluated CD155 expression on CD4^+^ T cells in the HIV and HC groups; a representative flow cytometry plot is presented in Figure 3A. Compared with those of the HC group, the expression levels of CD155 on CD4^+^ T cells were significantly higher in the HIV-infected group (*p* < 0.0001; Figure 3B). These data demonstrate that levels of CD155 are increased on CD4^+^ T cells after HIV infection and may influence NK cell function by binding with its receptors.

Given the increased expression of CD155 on CD4^+^ T cells, we next used PBMCs from HIV-infected individuals to evaluate the functional status of NK cells after blockage of CD155. For these experiments, anti-CD155 antibody was used to block the TIGIT ligand, CD155, and the IFN-γ production of NK cells subsequently evaluated. As we did not observe a statistically significant difference in the levels of IFN-γ production after CD155 inhibition (*p* = 0.1953; Figures 3C,D), our data suggest that the inhibitory effect of the TIGIT/CD155 axis on NK cell function in HIV-infected individuals cannot be reversed by blocking CD155. This might because CD155 can also be recognized by the activating receptor, CD226 (DNAM-1), which competes with TIGIT for binding to CD155 and delivers a positive co-stimulatory signal (10, 23). Hence, blockade of CD155 may also influence the CD226/CD155 axis.

### TIGIT is mainly expressed on CD226^+^ NK cells in HIV-infected individuals

**Figure 4:**
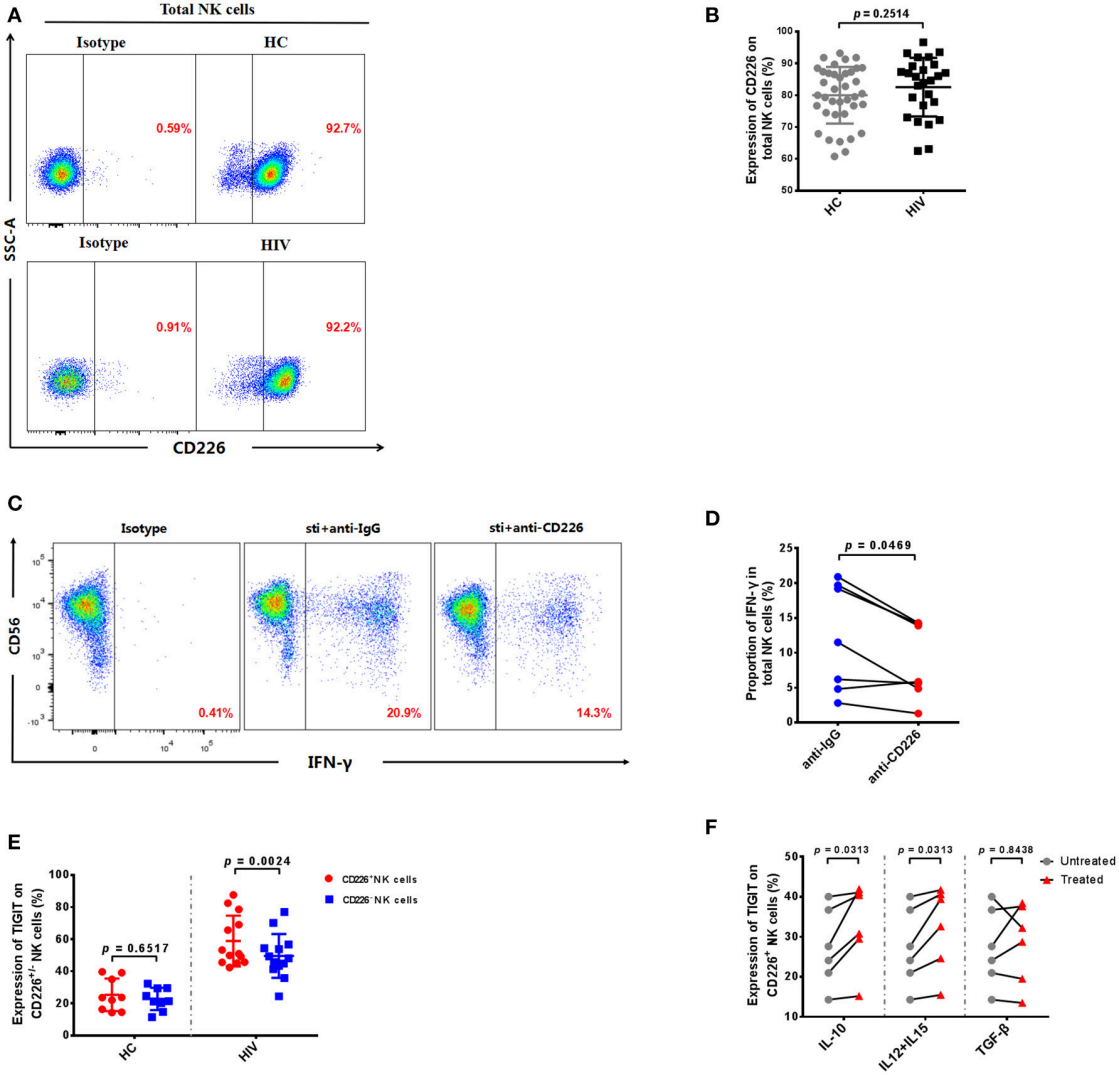
TIGIT is primarily expressed on CD226^+^ NK cells in HIV-infected individuals. **(A)** A representative flow cytometry plot showing the expression of CD226 on total NK cells in HIV-infected and HC individuals. **(B)** Comparison of the percentages of CD226^+^ NK cells in HIV-infected (*n* = 26) and HC (*n* = 38) groups. **(C)** A representative flow cytometry plot showing the proportion of IFN-γ producing NK cells after treatment with 20 μg/mL anti-human CD226 antibodies or purified mouse IgG (as a negative control) from HIV-infected individuals. The expression of IFN-γ was gated according to an isotype control. **(D)** Paired comparisons of the proportion of IFN-γ producing NK cells from HIV-infected individuals (*n* = 8) after treatment with 20 μg/mL anti-human CD155 antibodies or purified anti-mouse IgG (as a negative control). **(E)** Comparison of TIGIT expression on CD226^+^ NK cells in the HIV (*n* = 13) and HC (*n* = 9) groups. **(F)** Paired comparisons of the expression of TIGIT on CD226^+^ NK cells unstimulated or stimulated with rIL-10, rIL-12 + rIL-15, or rTGF-β. A Mann-Whitney *U* test was used for comparisons between two groups. A Wilcoxon matched-pairs signed-rank test was used for paired-group comparisons. *p* < 0.05 was considered significant.

Our experiments demonstrate that the expression levels of TIGIT and CD155 were significantly increased in HIV-infected individuals, and previous reports indicate that CD226 (DNAM-1) competes with TIGIT for binding to CD155, to deliver a positive co-stimulatory signal (10, 23). Therefore, we determined the expression of CD226 on NK cells in HIV-infected individuals. The result showed there was no significant difference in the expression of CD226 on NK cells between the HIV-infected and HC groups (*p* = 0.2514; Figures 4A,B). These data indicate that HIV infection does not affect CD226 expression on NK cells.

Since the expression of CD226 was not altered, we further investigated whether CD226 was able to stimulate NK cell function in HIV-infected individuals. We blocked CD226 with anti-CD226 antibodies, and then determined levels of IFN-γ production by NK cells using flow cytometry. The production of IFN-γ in NK cells was decreased after blockade of CD226 in HIV-infected individuals (*p* = 0.0469; Figures 4C,D). This result indicates that CD226 can still positively influence NK cell function during HIV infection.

Our data demonstrate that CD226 receptor levels are not altered and that CD226 can simulate NK cell function in HIV-infected individuals; therefore, it remains unclear why the function of NK cells in the HIV-infected group was decreased compared with those from the HC group. We further investigated whether the expression of TIGIT was associated with that of CD226 in HIV-infected NK cells. Interestingly, we found a higher level of TIGIT receptor expression on CD226^+^ NK cells in the HIV group (*p* = 0.0024; Figure 4E); however, the expression of TIGIT on CD226^+^ and CD226^−^ NK cells had no significantly different in the HC group (*p* = 0.6517; Figure 4E). These results show that the increased levels of TIGIT receptors in the HIV-infected group were mainly concentrated on the CD226^+^ NK cell population and could inhibit the role of CD226 in positive regulation, resulting in functional impairment of NK cells.

Given the significant increase of TIGIT expression on CD226^+^ NK cells in HIV-infected individuals, we further explored the factors leading to elevated TIGIT expression on this cell subset. Several cytokines can directly up-regulate negative checkpoint receptors on CD8^+^ T cells during chronic viral infections (34), and there are “cytokine storms” in the early stages of HIV infection, which result in abnormal cytokine levels (35); therefore, we stimulated NK cells from HIV-infected individuals using various cytokines. We found that stimulation with a combination of rIL-12 and rIL-15, or with rIL-10, led to significant increases in TIGIT expression on CD226^+^ NK cells from HIV-infected individuals (IL-12 + IL-15, *p* = 0.0313; IL-10, *p* = 0.0313; Figure 4F); however, no effects on these cells were observed on stimulation with rTGF-β (*p* = 0.8438; Figure 4F). These findings suggest that the expression of TIGIT on CD226^+^ NK cells from HIV-infected individuals may be due (at least in part) to the expansion of the peripheral cytokine milieu that occurs during HIV infection, which may render the majority of virus-specific NK cells susceptible to negative regulation.

### Inhibition of TIGIT can restore the function of NK cells in HIV-infected individuals

**Figure 5:**
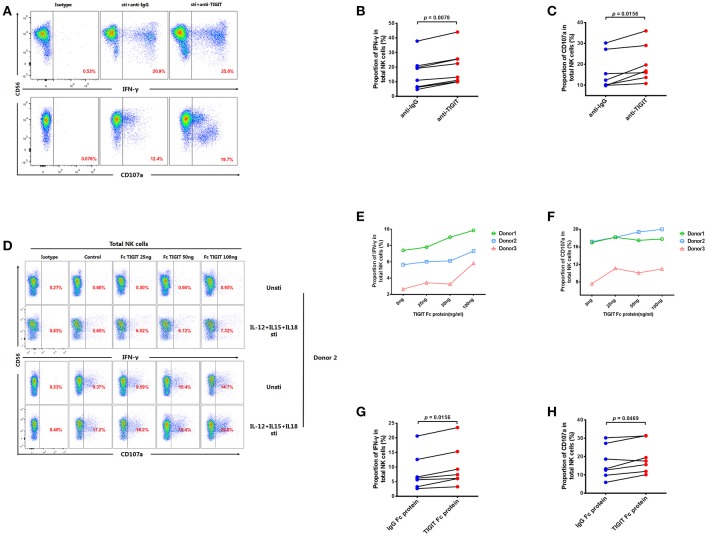
Blocking TIGIT can restore the function of NK cells in HIV-infected individuals. **(A)** A representative flow cytometry plot showing the proportion of IFN-γ producing NK cells after treatment with 5 μg/mL anti-human TIGIT antibodies or purified mouse IgG (as a negative control) from HIV-infected individuals. The expression of IFN-γ was gated according to an isotype control. **(B)** Paired comparisons of the proportions of IFN-γ producing NK cells after treatment with 5 μg/mL anti-human TIGIT antibodies or purified mouse IgG (as a negative control) from HIV-infected individuals (*n* = 8). **(C)** Paired comparisons of the proportion of CD107a expressed by NK cells after treatment with 5 μg/mL anti-human TIGIT antibodies or purified mouse IgG (as a negative control) from HIV-infected individuals (*n* = 7). **(D)** A representative flow cytometry plot demonstrating the effects of different doses of rTIGIT (0 (medium only), 20, 50, and 100 ng/mL) on IFN-γ and CD107a production by NK cells. **(E)** Proportion of IFN-γ^+^ NK cells from HIV-infected individuals after treatment with different concentrations of rTIGIT (0, 20, 50, and 100 ng/mL; *n* = 3). **(F)** Proportion of CD107a^+^ NK cells from HIV-infected individuals after treatment with different concentrations of rTIGIT (0, 20, 50, and 100 ng/mL; *n* = 3). **(G)** Paired comparisons of the proportion of IFN-γ producing NK cells after treatment with 50 ng/mL rTIGIT or rIgG from HIV-infected individuals (*n* = 7). **(H)** Paired comparisons of the proportion of CD107a-expressing NK cells after treatment with 50 ng/mL rTIGIT or rIgG from HIV-infected individuals (*n* = 7). Wilcoxon matched-pairs signed-rank tests were used for comparisons between paired groups. *p* < 0.05 was considered significant.

As our results indicate that TIGIT^+^ NK cells secrete less IFN-γ than TIGIT^−^ NK cells, we further explored whether NK cell function could be enhanced by treating PBMCs from HIV-infected individuals with anti-TIGIT antibody or rTIGIT, which are common reagents used for TIGIT blockade. After treatment with anti-TIGIT antibody for 24 h, both IFN-γ production and degranulation (CD107a) were significantly enhanced compared with controls (IFN-γ, *p* = 0.0078; CD107a, *p* = 0.0156; Figures 5A–C). Next, we evaluated the effects of different concentrations of rTIGIT (0, 25, 50, and 100 ng/mL) on NK cell function. The results demonstrated that higher concentrations of rTIGIT led to dose-dependent restoration of NK cell function (Figures 5D–F). To evaluate the effects on NK cells from HIV-infected individuals, an intermediate concentration of rTIGIT (50 ng/mL) was chosen. The results showed that levels of IFN-γ production were significantly increased after blockade using rTIGIT (*p* = 0.0156; Figure 5G). Similarly, enhanced degranulation, indicated by elevated CD107a expression, was observed after treatment with rTIGIT (*p* = 0.0469; Figure 5H). Together, these results indicate that both anti-TIGIT antibody and rTIGIT were able to reverse the inhibitory effect of TIGIT on NK cell function.

## Discussion

TIGIT has a profound impact in a variety of diseases. In autoimmune diseases, TIGIT has a protective role in rheumatoid arthritis, multiple sclerosis, and type 1 diabetes; however, TIGIT expression levels are substantially reduced in these diseases (36, 37). Conversely, levels of TIGIT are elevated in chronic infectious diseases and cancers, and negatively regulate anti-infection and anti-tumor responses (38, 39). Nevertheless, the effect of TIGIT on the functional responses of NK cells in HIV infection has not been previously investigated. Elucidation of the role of TIGIT on NK cells in this context may assist understanding of the dysfunctional immunobiology of these cells in HIV infection and provide a route toward their functional restoration. Therefore, it is necessary to determine the effects of TIGIT on NK cells in the context of HIV infection.

In this investigation, we dissected TIGIT expression on NK cells in HIV-infected individuals for the first time. We demonstrate that the expression of TIGIT on NK cells is significantly higher in HIV-infected individuals than controls and identify a correlation between TIGIT expression and HIV disease progression. Similarly, the expression of TIGIT on CD8^+^ T cells is also reported to be higher after HIV infection and to be associated with disease progression (17). The reason for the increased TIGIT levels on NK cells during HIV infection remains unknown; however, it may be related to activation of the immune system. Then we performed a comprehensive analysis of TIGIT inhibitory receptors on four NK cell subsets and found that the CD56^dim^CD16^−^, CD56^−^CD16^+^ and CD56^dim^CD16^+^ subsets had higher TIGIT expression in HIV-infected individuals. CD56^dim^CD16^+^ and CD56^dim^CD16^−^ subsets are cytotoxic cells and also produce IFN-γ to fight with virus (40–43). Higher expression of TIGIT on these subsets could impair their functions of controlling HIV infection. CD56^−^CD16^+^ subset up-regulated during HIV infection is known to be anergic (44, 45), the increased expression of TIGIT may explain the reason for its dysfunction in HIV infection. However, we did not observe an increased expression of TIGIT on CD56^bright^ subset, and it seems that the immature subset CD56^bright^ NK cells are more resistant to the expression of TIGIT, and their function might be less influenced. The related mechanisms are required for further investigation.

According to previous studies, T cell function is suppressed by TIGIT over-expression in HIV infection (17, 46); therefore, we hypothesized that expression of TIGIT may also reduce NK cell function in HIV-infected individuals. We observed a significant reduction in the production of IFN-γ by TIGIT^+^ NK cells from the HIV-infected group, and an inverse correlation between the percentage of TIGIT^+^ NK cells and that of IFN-γ^+^ NK cells, demonstrating that TIGIT has a negative regulatory function on NK cells during HIV infection. These data are supported by the findings from studies in mouse NK cells, where over-expression of TIGIT inhibits NK cell function (21, 47). The mechanism of TIGIT suppression of NK cell function has also been studied in NK cell lines, where TIGIT was shown to directly generate inhibitory signals through its ITIM domain, leading to inhibitory effects by binding with CD155 (13, 48). Further, the phosphorylated ITT-like motif of TIGIT can combine with β-arrestin2, which recruits SHIP1 to limit nuclear factor-κB (NF-κB) signaling, leading to substantial reduction of IFN-γ production by NK cells (48–50). In addition, fusion of a TIGIT tail to SHIP1 can block mitogen-activated protein kinase and phosphoinositide 3-kinase pathways, potentially mediating reduced cytokine production and survival, respectively, in NK cells (48–50). The exact details of these pathways remain unclear, hence the mechanisms underlying TIGIT function require further investigation.

The expression of the TIGIT ligand, CD155, on CD4^+^ T cells was found to be higher in HIV-infected individuals than healthy controls; however, our results indicate that blockage of CD155 does not restore IFN-γ production by NK cells. In a previous study, Pfeiffer's team showed that blockade of CD155 resulted in decreased lysis of human hepatoblastoma cells (51), while Baofu's group demonstrated that blocking CD155 could enhance the proliferation of healthy CIK cells (52). In addition, Kraus' team found that the absence of CD112 or CD155 in kidney allografts did not significantly influence renal function (53). These discordant results may be attributable to CD155 biological function, which can combine with either TIGIT or CD226; hence, the results of CD155 blockage may be influenced by the ratio of TIGIT to CD226 expression levels.

The CD226 receptor, which competes with TIGIT for the same ligands, can promote the cytotoxic and anti-tumor responses of mouse NK cells (54). Moreover, Peng et al. (55) suggested that lower levels of CD226^+^ NK cells may contribute to tumor immune escape. Here we investigated the expression of the CD226 receptor in HIV-infected individuals; however, we did not identify any significant difference in CD226 levels on NK cells from the HIV-infected and HC groups. Furthermore, we found that NK cell function was impaired by CD226 blockade in HIV-infected individuals, suggesting that the CD226 receptor may have a positive effect on NK cell activity. However, in HIV-infected individuals, the function of NK cells was reduced, indicating that, although they are expressed at normal levels, CD226 receptors were unable to function in their activating role; therefore, we explored the reason for CD226 receptor function impairment. We found that, in HIV-infected individuals, the TIGIT receptor was specifically expressed on CD226^+^ NK cells. Since TIGIT has a much higher affinity than CD226 for binding with CD155, the interaction between CD226 and CD155 can be inhibited by TIGIT in a dose-dependent manner (18, 48). Furthermore, TIGIT can also directly combine with CD226 in *cis*, and interfere with its homodimerization (15, 56, 57). These data demonstrate that the role of CD226 as an activating receptor on NK cells can be repressed by over-expression of TIGIT in HIV-infected individuals, leading to reduced activation of their NK cells.

During the early stages of HIV infection, levels of numerous plasma proteins and activation markers, including TGF-β, IL-15, IL-12, and IL-10, among others, are reported to alter substantially, assisting virus dissemination and the immune inflammatory reaction (58, 59). A previous study demonstrated that IL-2 and IL-15 can increase the expression of TIGIT on CD8^+^ T cells in HIV-infected individuals (17), and we further explored whether these cytokines lead to higher expression of TIGIT on CD226^+^ NK cells during HIV infection. We assessed that the expression of TIGIT on CD226^+^ NK cells was up-regulated by the cytokines IL-10, and IL-12 + IL-15, but not TGF-β, in HIV-infected individuals, implying that a disturbed cytokine milieu mediates the over-expression of TIGIT to inhibit activation of CD226^+^ NK cells during HIV infection. Additionally, our data indicate that inhibition of TIGIT can restore the function of NK cells from HIV-infected individuals, implying that both anti-TIGIT antibodies and rTIGIT can serve as approaches to enhance NK cell function.

Overall, this study demonstrates that the proportion of TIGIT are higher on NK cells, and suppress the function of NK cells, in HIV-infected individuals, and that they are associated with HIV disease progression. Furthermore, TIGIT expression is specifically elevated on CD226^+^ NK cells, and rIL-10, or rIL-12 + rIL-15, can induce TIGIT expression. High levels of TIGIT expression can inhibit IFN-γ production by NK cells, while blockade of TIGIT can restore their function, suggesting targeting of TIGIT as a potential immune therapeutic strategy for treatment of HIV infection.

## Limitations

There was one limitation in our study which should be considered when interpreting our results. We excluded non-NK cells in our total NK cells by gating the lymphocytes according to forward scatter/side scatter properties (FSC/SSC) and CD3, CD56, CD16 markers. There might possibly be about 1% non-NK cells in our total NK cells. It would be the better way to identify the total NK cells with the additional marker CD14, CD19, CD11c, CD123, M-DC8 and HLA-DR antibodies to exclude non-NK cells.

## Ethics approval and consent to participate

The Medical Science Research Ethics Committee of the First Affiliated Hospital of China Medical University (Shenyang, China) approved the study protocol (KELUNSHEN [2011] number 36). Our study was conducted according to the principles enshrined in the Declaration of Helsinki. Written informed consent to take part in the study was obtained from all participants.

## Author contributions

YJ and HS designed the experiments, interpreted the data and revised the manuscript. XY, TL, and ZW carried out the NK cell experiments and analyzed the data. XY and JL wrote the manuscript. ZZ and YF carried out CD4^+^ T cell counts. MM and SF participated in the experiments to detect IFN-γ production. XH participated in measurement of HIV viral load. QH, HD, and JX carried out the epidemiological study and helped to recruit study participants. We have listed substantial, direct, and intellectual contributions to the work. All authors have read the manuscript and approved it for publication.

### Conflict of interest statement

The authors declare that the research was conducted in the absence of any commercial or financial relationships that could be construed as a potential conflict of interest.

## Supplementary material

Figure S1 Gating strategy used to identify total natural killer (NK) cells and NK cell subsets in HC group. Single cells were gated using the forward scatter area (FSA) and forward scatter height (FSH), then live cells were gated by Live/Dead (BV510) staining. Lymphocytes were gated according to forward scatter/side scatter properties (FSC/SSC). Total NK cells were identified from CD3-negative lymphocytes by their expression of CD16 and/or CD56. The four NK cell subsets identified were CD3^−^CD56^bright^CD16^−/+^, CD3^−^CD56^dim^CD16^+^, CD3^−^CD56^dim^CD16^−^, and CD3^−^CD56^−^CD16^+^. All the plots were based on a HC individual.

Figure S2 The proportions of four NK cell subsets in the HIV-infected and HC groups. Comparisons of the percentages of different NK cell subsets between HC (*n* = 26) and HIV-infected (*n* = 38) groups. A Mann-Whitney U test was used for comparisons between two groups. Error bars indicate median and interquartile range. *p* < 0.05 was considered significant.

Figure S3 The expression of TIGIT on NK cell subsets and correlation with the CD4^+^ T cell counts. **(A)** Representative flow cytometry plots showing the percentages of TIGIT on four NK cell subsets (CD3^−^CD56^bright^CD16^−/+^, CD3^−^CD56^dim^CD16^+^, CD3^−^CD56^dim^CD16^−^, and CD3^−^CD56^−^CD16^+^) in the HC and HIV groups. The expression of TIGIT was gated according to an isotype control. **(B)** Comparisons of the percentages of TIGIT expression among different NK cell subsets in the HC group (*n* = 26). **(C)** Comparisons of the percentage of TIGIT expression among different NK cell subsets in the HIV-infected group (*n* = 38). **(D)** Comparisons of the percentages of TIGIT on different NK cell subsets between HC (*n* = 26) and HIV-infected (*n* = 38) groups. **(E)** Analysis of the correlation between TIGIT expression on CD56^−^CD16^+^ NK cells and absolute CD4^+^ T cell counts (cells/mm^3^) at the same sampling time (*n* = 53). **(F)** Analysis of the correlation between TIGIT expression on CD56^bright^CD16^−/+^ NK cells and absolute CD4^+^ T cell counts (cells/mm^3^) at the same sampling time (*n* = 53). **(G)** Analysis of the correlation between TIGIT expression on CD56^dim^CD16^+^ NK cells and absolute CD4^+^ T cell counts (cells/mm^3^) at the same sampling time (*n* = 53). **(H)** Analysis of the correlation between TIGIT expression on CD56^dim^CD16^−^ NK cells and absolute CD4^+^ T cell counts (cells/mm^3^) at the same sampling time (*n* = 53). The Mann-Whitney test was used for comparisons between two groups, and the Kruskal-Wallis test for comparisons among the four groups. Error bars indicate median and interquartile range. *p* < 0.05 was considered significant.
