# Supplementary figures and images for: Expression of the Inhibitory Receptor TIGIT Is Up-Regulated Specifically on NK Cells With CD226 Activating Receptor From HIV-Infected Individuals
Source: Front Immunol. 2018 Oct 10;9:2341. doi: 10.3389/fimmu.2018.02341 (PMC6192288; doi:10.3389/fimmu.2018.02341)

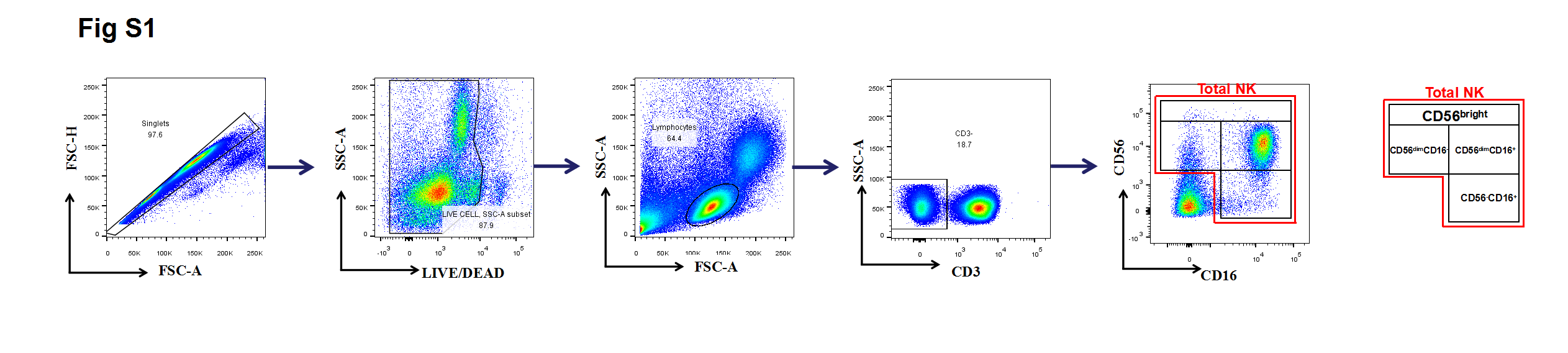

Supplement: Figure S1 — Gating strategy used to identify total natural killer (NK) cells and NK cell subsets in HC group. Single cells were gated using the forward scatter area (FSA) and forward scatter height (FSH), then live cells were gated by Live/Dead (BV510) staining. Lymphocytes were gated according to forward scatter/side scatter properties (FSC/SSC). Total NK cells were identified from CD3-negative lymphocytes by their expression of CD16 and/or CD56. The four NK cell subsets identified were CD3−CD56brightCD16−/+, CD3−CD56dimCD16+, CD3−CD56dimCD16−, and CD3−CD56−CD16+. All the plots were based on a HC individual. [file Image_1.TIF]

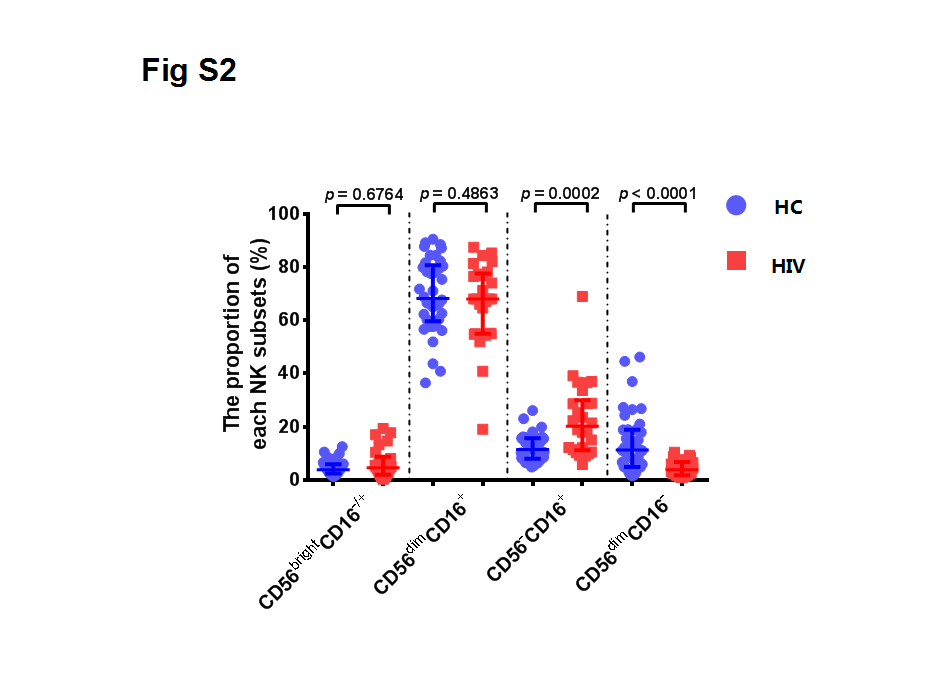

Supplement: Figure S2 — The proportions of four NK cell subsets in the HIV-infected and HC groups. Comparisons of the percentages of different NK cell subsets between HC (n = 26) and HIV-infected (n = 38) groups. A Mann-Whitney U test was used for comparisons between two groups. Error bars indicate median and interquartile range. p < 0.05 was considered significant. [file Image_2.TIF]

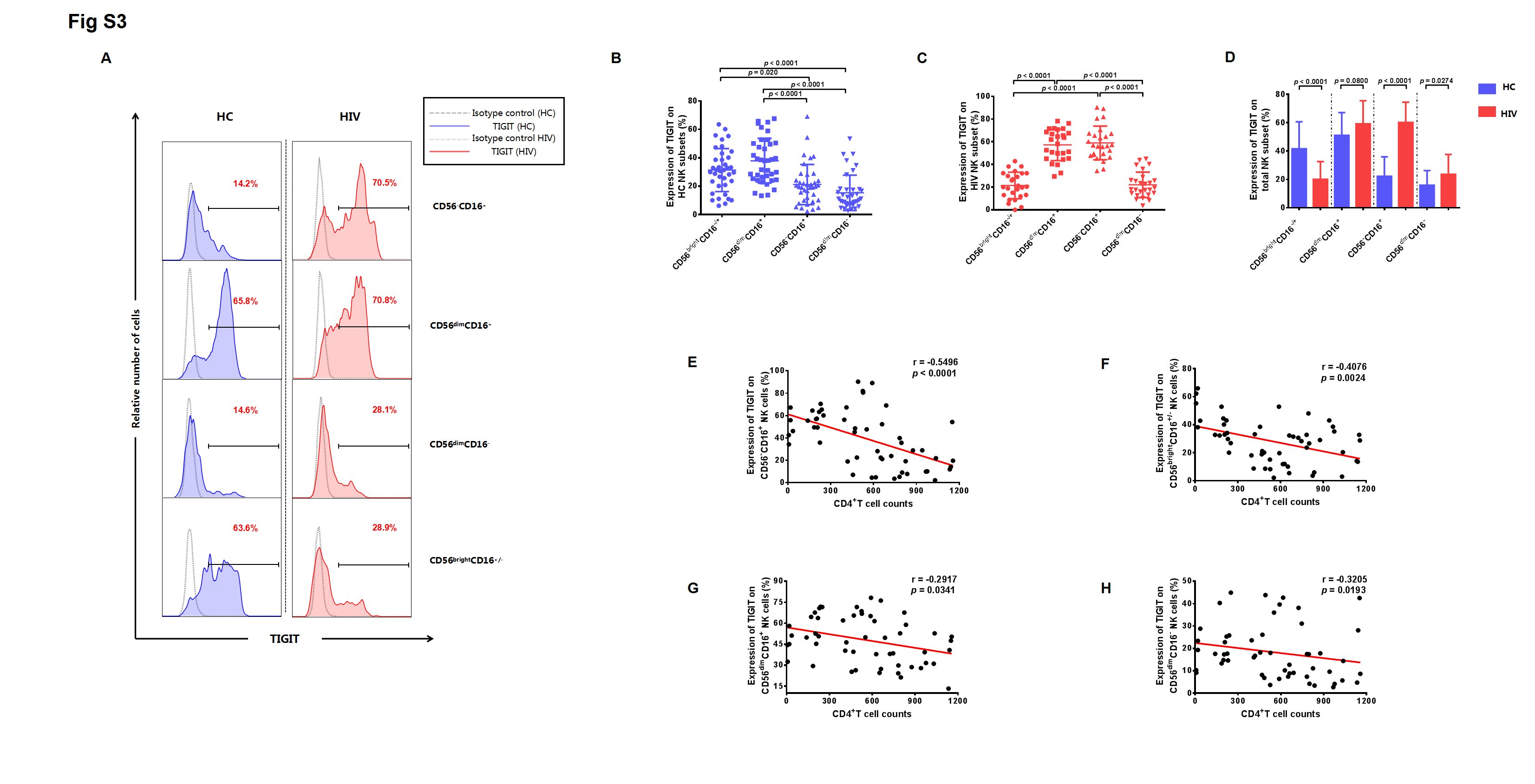

Supplement: Figure S3 — The expression of TIGIT on NK cell subsets and correlation with the CD4+ T cell counts. (A) Representative flow cytometry plots showing the percentages of TIGIT on four NK cell subsets (CD3−CD56brightCD16−/+, CD3−CD56dimCD16+, CD3−CD56dimCD16−, and CD3−CD56−CD16+) in the HC and HIV groups. The expression of TIGIT was gated according to an isotype control. (B) Comparisons of the percentages of TIGIT expression among different NK cell subsets in the HC group (n = 26). (C) Comparisons of the percentage of TIGIT expression among different NK cell subsets in the HIV-infected group (n = 38). (D) Comparisons of the percentages of TIGIT on different NK cell subsets between HC (n = 26) and HIV-infected (n = 38) groups. (E) Analysis of the correlation between TIGIT expression on CD56−CD16+ NK cells and absolute CD4+ T cell counts (cells/mm3) at the same sampling time (n = 53). (F) Analysis of the correlation between TIGIT expression on CD56brightCD16−/+ NK cells and absolute CD4+ T cell counts (cells/mm3) at the same sampling time (n = 53). (G) Analysis of the correlation between TIGIT expression on CD56dimCD16+ NK cells and absolute CD4+ T cell counts (cells/mm3) at the same sampling time (n = 53). (H) Analysis of the correlation between TIGIT expression on CD56dimCD16− NK cells and absolute CD4+ T cell counts (cells/mm3) at the same sampling time (n = 53). The Mann-Whitney test was used for comparisons between two groups, and the Kruskal-Wallis test for comparisons among the four groups. Error bars indicate median and interquartile range. p < 0.05 was considered significant. [file Image_3.TIF]
